# Supplementary material for: Identification and functional analysis of non-coding regulatory small RNA FenSr3 in Bacillus amyloliquefaciens LPB-18
Source: PeerJ. 2023 May 15;11:e15236. doi: 10.7717/peerj.15236 (PMC10194069; doi:10.7717/peerj.15236)
Supplement: Supplemental Information 4 [file peerj-11-15236-s004.zip › KO/CK-vs-T1_map/map01130.html]

KEGG PATHWAY: Biosynthesis of antibiotics - Reference pathway


|  |  |
| --- | --- |
| **Biosynthesis of antibiotics - Reference pathway** |  |

[
Pathway menu
| Organism menu
| Pathway entry
|

Hide module list

| User data mapping
|

Image (png) file

]

|  |  |  |
| --- | --- | --- |
| Reference pathway | 100% 70% 35% | 图片下载 |

- **Category title**

  

- **KEGG module**

- Carbohydrate and lipid metabolism
  - Other carbohydrate metabolism
    - M00793 dTDP-L-rhamnose biosynthesis- M00794 dTDP-6-deoxy-D-allose biosynthesis
      - M00795 dTDP-beta-L-noviose biosynthesis
      - M00796 dTDP-D-mycaminose biosynthesis
      - M00797 dTDP-D-desosamine biosynthesis
      - M00798 dTDP-L-mycarose biosynthesis
      - M00799 dTDP-L-oleandrose biosynthesis
      - M00800 dTDP-L-megosamine biosynthesis
      - M00801 dTDP-L-olivose biosynthesis
      - M00802 dTDP-D-forosamine biosynthesis
      - M00803 dTDP-D-angolosamine biosynthesis
  - Terpenoid backbone biosynthesis
    - M00849 C5 isoprenoid biosynthesis, mevalonate pathway, archaea- M00364 C10-C20 isoprenoid biosynthesis, bacteria
  - Sterol biosynthesis
    - M00102 Ergocalciferol biosynthesis
- Nucleotide and amino acid metabolism
  - Aromatic amino acid metabolism
    - M00022 Shikimate pathway, phosphoenolpyruvate + erythrose-4P => chorismate
- Secondary metabolism
  - Biosynthesis of secondary metabolites
    - M00672 Penicillin biosynthesis, aminoadipate + cycteine + valine => penicillin- M00673 Cephamycin C biosynthesis, aminoadipate + cycteine + valine => cephamycin C
      - M00675 Carbapenem-3-carboxylate biosynthesis, pyrroline-5-carboxylate + malonyl-CoA => carbapenem-3-carboxylate
      - M00736 Nocardicin A biosynthesis, L-pHPG + arginine + serine => nocardicin A
      - M00674 Clavaminate biosynthesis, arginine + glyceraldehyde-3P => clavaminate
      - M00773 Tylosin biosynthesis, methylmalonyl-CoA + malonyl-CoA => tylactone => tylosin
      - M00774 Erythromycin biosynthesis, propanoyl-CoA + methylmalonyl-CoA => deoxyerythronolide B => erythromycin A/B
      - M00775 Oleandomycin biosynthesis, malonyl-CoA + methylmalonyl-CoA => 8,8a-deoxyoleandolide => oleandomycin
      - M00776 Pikromycin/methymycin biosynthesis, methylmalonyl-CoA + malonyl-CoA => narbonolide/10-deoxymethynolide => pikromycin/methymycin
      - M00777 Avermectin biosynthesis, 2-methylbutanoyl-CoA/isobutyryl-CoA => 6,8a-Seco-6,8a-deoxy-5-oxoavermectin 1a/1b aglycone => avermectin A1a/B1a/A1b/B1b
      - M00778 Type II polyketide backbone biosynthesis, acyl-CoA + malonyl-CoA => polyketide
      - M00779 Dihydrokalafungin biosynthesis, octaketide => dihydrokalafungin
      - M00780 Tetracycline/oxytetracycline biosynthesis, pretetramide => tetracycline/oxytetracycline
      - M00823 Chlortetracycline biosynthesis, pretetramide => chlortetracycline
      - M00781 Nogalavinone/aklavinone biosynthesis, deoxynogalonate/deoxyaklanonate => nogalavinone/aklavinone
      - M00782 Mithramycin biosynthesis, 4-demethylpremithramycinone => mithramycin
      - M00783 Tetracenomycin C/8-demethyltetracenomycin C biosynthesis, tetracenomycin F2 => tetracenomycin C/8-demethyltetracenomycin C
      - M00784 Elloramycin biosynthesis, 8-demethyltetracenomycin C => elloramycin A
      - M00786 Fumitremorgin alkaloid biosynthesis, tryptophan + proline => fumitremorgin C/A
      - M00789 Rebeccamycin biosynthesis, tryptophan => rebeccamycin
      - M00790 Pyrrolnitrin biosynthesis, tryptophan => pyrrolnitrin
      - M00805 Staurosporine biosynthesis, tryptophan => staurosporine
      - M00808 Violacein biosynthesis, tryptophan => violacein
      - M00814 Acarbose biosynthesis, sedoheptulopyranose-7P => acarbose
      - M00815 Validamycin A biosynthesis, sedoheptulopyranose-7P => validamycin A
      - M00785 Cycloserine biosynthesis, arginine/serine => cycloserine
      - M00787 Bacilysin biosynthesis, prephenate => bacilysin
      - M00788 Terpentecin biosynthesis, GGAP => terpentecin
      - M00819 Pentalenolactone biosynthesis, farnesyl-PP => pentalenolactone
      - M00848 Aurachin biosynthesis, anthranilate => aurachin A
      - M00824 9-membered enediyne core biosynthesis, malonyl-CoA => 3-hydroxyhexadeca-4,6,8,10,12,14-hexaenoyl-ACP => 9-membered enediyne core
      - M00825 10-membered enediyne core biosynthesis, malonyl-CoA => 3-hydroxyhexadeca-4,6,8,10,12,14-hexaenoyl-ACP => 10-membered enediyne core
      - M00826 C-1027 benzoxazolinate moiety biosynthesis, chorismate => benzoxazolinyl-CoA
      - M00827 C-1027 beta-amino acid moiety biosynthesis, tyrosine => 3-chloro-4,5-dihydroxy-beta-phenylalanyl-PCP
      - M00828 Maduropeptin beta-hydroxy acid moiety biosynthesis, tyrosine => 3-(2-chloro-3-hydroxy-4-methoxyphenyl)-3-hydroxypropanoyl-PCP
      - M00829 3,6-Dimethylsalicylyl-CoA biosynthesis, malonyl-CoA => 6-methylsalicylate => 3,6-dimethylsalicylyl-CoA
      - M00830 Neocarzinostatin naphthoate moiety biosynthesis, malonyl-CoA => 2-hydroxy-5-methyl-1-naphthoate => 2-hydroxy-7-methoxy-5-methyl-1-naphthoyl-CoA
      - M00831 Kedarcidin 2-hydroxynaphthoate moiety biosynthesis, malonyl-CoA => 3,6,8-trihydroxy-2-naphthoate => 3-hydroxy-7,8-dimethoxy-6-isopropoxy-2-naphthoyl-CoA
      - M00832 Kedarcidin 2-aza-3-chloro-beta-tyrosine moiety biosynthesis, azatyrosine => 2-aza-3-chloro-beta-tyrosyl-PCP
      - M00834 Calicheamicin orsellinate moiety biosynthesis, malonyl-CoA => orsellinate-ACP => 5-iodo-2,3-dimethoxyorsellinate-ACP
      - M00833 Calicheamicin biosynthesis, calicheamicinone => calicheamicin
      - M00835 Pyocyanine biosynthesis, chorismate => pyocyanine
      - M00837 Prodigiosin biosynthesis, L-proline => prodigiosin
      - M00838 Undecylprodigiosin biosynthesis, L-proline => undecylprodigiosin
